# Prevalence and Predictors of Sepsis Death in Patients With Chronic Heart Failure and Reduced Left Ventricular Ejection Fraction

**DOI:** 10.1161/JAHA.118.009684

**Published:** 2018-10-04

**Authors:** Andrew M. N. Walker, Michael Drozd, Marlous Hall, Peysh A. Patel, Maria Paton, Judith Lowry, John Gierula, Rowenna Byrom, Lorraine Kearney, Robert J. Sapsford, Klaus K. Witte, Mark T. Kearney, Richard M. Cubbon

**Affiliations:** ^1^ Leeds Institute of Cardiovascular and Metabolic Medicine The University of Leeds United Kingdom; ^2^ Department of Cardiology Leeds General Infirmary Leeds Teaching Hospitals NHS Trust Leeds United Kingdom

**Keywords:** heart failure, morbidity/mortality, sepsis, Heart Failure, Mortality/Survival

## Abstract

**Background:**

Noncardiovascular death is increasingly common in people with chronic heart failure (CHF), yet its causes remain poorly characterized. We aimed to define the prevalence of sepsis death in people with CHF and to ascertain its risk marker profile.

**Methods and Results:**

We conducted a prospective cohort study of 1802 patients with CHF and left ventricular ejection fraction ≤45% attending CHF clinics in 4 United Kingdom hospitals between 2006 and 2014. Mode of death was defined over a 10.3‐year follow‐up period (mean 4 years). Competing risk regression defined mode‐specific hazard ratios for sepsis, other noncardiovascular, progressive heart failure, and sudden cardiac death in relation to established heart failure prognostic markers. Of 737 deaths, 173 (23.5%) were due to sepsis; respiratory tract infections accounted for 69.9% (n=121) of these events. Those who died from sepsis were older, had higher platelet counts, and had a higher prevalence of chronic obstructive pulmonary disease than those who died from other causes. Sepsis death was independently associated with older age (hazard ratio=1.05; 95% confidence interval 1.03‐1.07), greater prevalence of chronic obstructive pulmonary disease (2.43; 1.74‐3.40), male sex (1.73; 1.16‐2.60), lower log serum vitamin D (0.68; 0.49‐0.95), and higher platelet count (1.002; 1.000‐1.005) than nonsepsis death. Established heart failure prognostic markers exhibited different patterns of association with sepsis death, other noncardiovascular death, progressive heart failure death, and sudden cardiac death.

**Conclusions:**

Sepsis is a major contributor to death in people with CHF and has a different risk marker profile from other modes of death, suggesting that it may be amenable to targeted preventative strategies.

**Clinical Perspective**

**What Is New?**

- Sepsis accounts for almost one quarter of deaths in people with heart failure.
- Advancing age, chronic obstructive pulmonary disease, male sex, lower serum vitamin D, and increasing platelet count are independent risk markers for sepsis death.
- Sepsis death has a risk marker profile that is distinct from progressive heart failure death, sudden cardiac death, and other noncardiovascular death.

**What Are the Clinical Implications?**

- Strategies to improve outcomes in heart failure populations will need to consider prevention, identification, and management of sepsis.
- Patients at high risk of sepsis death can be identified using simple clinical data.

## Introduction

Advances in the management of cardiovascular disease have been associated with a progressive rise in the prevalence of chronic heart failure (CHF).^1^ Similar advances in heart failure care have been associated with greater survival of people who develop heart failure.^2^, ^3^, ^4^ Importantly, the reduced mortality of people with heart failure has also been associated with an increasing contribution of noncardiovascular modes of death.^2^, ^5^, ^6^ Sepsis has been identified as a contributor to noncardiovascular death^6^, ^7^ and hospitalization^8^, ^9^ in mixed heart‐failure populations including people with preserved and reduced left ventricular ejection fraction (LVEF). However, which people are at greatest risk of sepsis death and whether a sepsis‐specific risk marker profile exists remain uncertain. We aimed to define the prevalence of sepsis death in ambulant CHF patients with reduced LVEF, define risk markers for sepsis death, and then contrast the risk marker profile of sepsis death with those of other competing modes.

## Methods

### Data Availability

The data, analytic methods, and study materials will not be made freely available to other researchers for purposes of reproducing the results or replicating the procedure because the complete study data set contains potentially identifying data; however, data will be made available by the corresponding author to other researchers who have appropriate ethical approval and data protection arrangements.

### Study Design

As described in our earlier publications, we conducted a prospective cohort study with the predefined aim of identifying prognostic markers in patients with CHF and LVEF, receiving contemporary evidence‐based therapy^10^, ^11^; the aim of this analysis of the cohort study was to specifically focus on sepsis‐related death. Inclusion in the study required the presence of stable signs and symptoms of CHF for at least 3 months, age ≥18 years, and LVEF ≤45% on transthoracic echocardiography. Between June 2006 and December 2014, consecutive patients attending specialist cardiology clinics (secondary and tertiary referral) in 4 UK hospitals were approached, and 1802 patients provided written informed consent. The Leeds West Research Ethics Committee gave ethical approval, and the investigation conformed to the principles outlined in the Declaration of Helsinki.

As described previously,^11^ details of medical history were collected at recruitment, and symptomatic status was defined using the New York Heart Association classification. Venous blood was collected at study recruitment for measurement of electrolyte concentrations and assessment of renal function and hematological parameters; these tests were performed in the local hospital chemical pathology laboratories. Estimated glomerular filtration rate was calculated using the Modification of Diet in Renal Disease method, as detailed in our earlier description of this cohort study.^11^ Two‐dimensional echocardiography was performed according to The American Society of Echocardiography recommendations. Resting heart rate was measured using 12‐lead ECGs. Prescribed doses of loop diuretics, angiotensin‐converting enzyme inhibitors, angiotensin receptor blockers, and β‐blockers were collected at study recruitment. Total daily doses of β‐blocker, angiotensin‐converting enzyme inhibitors (or angiotensin receptor blocker if used instead of angiotensin‐converting enzyme inhibitors), and loop diuretic were expressed relative to the maximal licensed dose of bisoprolol, ramipril, and furosemide, respectively, as previously published.^11^ Receipt of cardiac resynchronization therapy or an implantable cardioverter‐defibrillator was assessed during the 6‐month period after recruitment.

### Mortality Classification

All patients were registered with the UK Office of Population Censuses and Surveys, which provided details of time of death, with a final censorship date of May 8, 2016. Classification criteria for the mode of death were defined before the study commenced, as previously reported.^2^ Importantly, mode of death describes the broad mechanism of death but is different from attributing a specific cause of death (for example, a progressive heart failure death could have many different causes, such as dilated cardiomyopathy or ischemic heart disease), aiming to describe how people die and how overall therapeutic strategies may need to evolve. In brief, all deaths were evaluated by at least 2 clinicians who reviewed death certificates, autopsy findings, and medical records, as appropriate; when insufficient information was available, the mode of death was deemed unclassifiable. Mode of death was classified as: (1) sudden cardiac, if occurring within 1 hour of a change in symptoms, during sleep, or while the patient was unobserved; (2) progressive heart failure, if occurring after a documented period of symptomatic or hemodynamic deterioration; (3) other cardiovascular death, if not occurring suddenly or in association with progression of heart failure; and (4) noncardiovascular death. Noncardiovascular death was subclassified by the evaluating clinicians as sepsis death if preceded by deteriorating symptoms, signs (eg, pyrexia, tachycardia, hypotension, tachypnea, confusion) and laboratory indices of sepsis (eg, elevated inflammatory markers, possibly with microbiological, serological, and/or imaging evidence), resulting in treatment with antimicrobial therapy.

### Statistical Analysis

All statistical analyses were performed using IBM SPSS statistics version 21 (IBM Corporation, Armonk, NY) and Stata MP64 version 14 (Statacorp LLC, College Station, TX). Continuous data are presented as mean (standard error of the mean) or median (interquartile range) for normal and nonnormally distributed variables, respectively, and categorical data are shown as percentage (number). Groups were compared using Student t tests or ANOVA for normally distributed continuous data, Mann‐Whitney U tests for nonnormally distributed continuous data, and Pearson χ^2^ tests for categorical data. All tests were 2‐sided, and statistical significance was defined as *P*<0.05.

Unadjusted and adjusted cause‐specific hazard ratios were estimated using the Fine and Gray competing risk regression model and by treating sepsis death as the event of interest and all other causes of death as competing events.^12^ Adjusted models included the following variables: age at study recruitment, male sex, diabetes mellitus, chronic obstructive pulmonary disease (COPD), ischemic CHF etiology, New York Heart Association class, heart rate, QRS interval, hemoglobin, lymphocyte count, neutrophil count, platelet count, sodium, estimated glomerular filtration rate, albumin, vitamin D (natural log transformed), LVEF, ramipril dose, bisoprolol dose, furosemide dose, implantable cardioverter‐defibrillator, and cardiac resynchronization therapy (all selected based on prior associations with mortality in people with heart failure). To determine the risk profiles of progressive heart failure and sudden cardiac death, 2 subsequent competing risk models in which each respective mode of death was treated as the event of interest in turn were fitted to the data with the same adjustment variables. Finally, an adjusted Cox proportional hazards model was fitted to determine the risk profile associated with all‐cause mortality. The relative importance of variables associated with each cause of death was compared by ranking the percentage contribution of the variable‐specific Wald test out of the global model Wald test and displayed as a heat map.

Multiple imputation by chained equations was used to produce 50 imputed data sets to minimize potential bias due to missing data (Data S1). Pooled estimates and accompanying 95% confidence intervals (CIs) over each imputed data set were generated according to Rubin rules for all unadjusted and adjusted regression models. Notably, there were minimal missing data for most variables, other than vitamin D (30.5% missing), which is due to the late inclusion of this in our prospective data collection protocol as a result of data implicating this in heart failure outcomes.^13^

## Results

**Figure 1:**
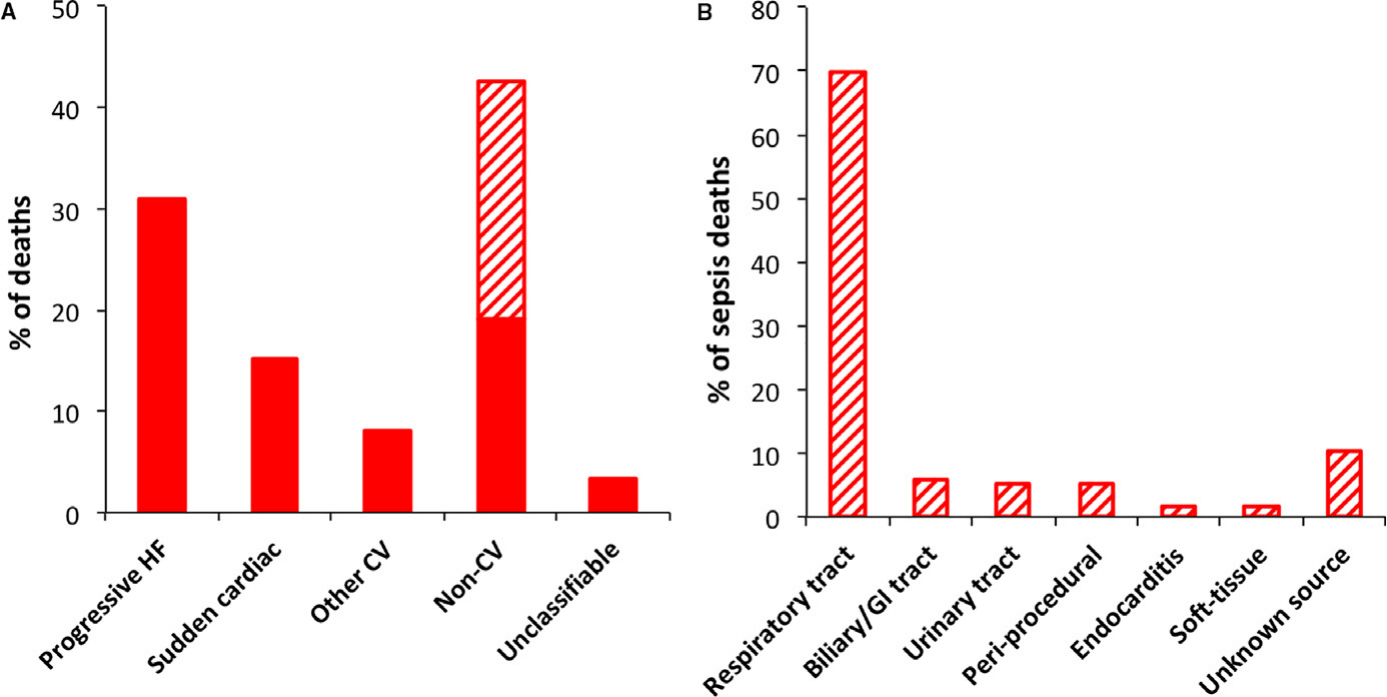
Sepsis death in relation to other common modes of death. A, Relative contribution of modes of death to overall mortality, with sepsis (represented by the hatched region) accounting for over half of noncardiovascular deaths. B, Relative contribution of primary sources of sepsis to overall sepsis deaths. CV indicates cardiovascular; GI, gastrointestinal; HF, heart failure.

**Figure 2:**
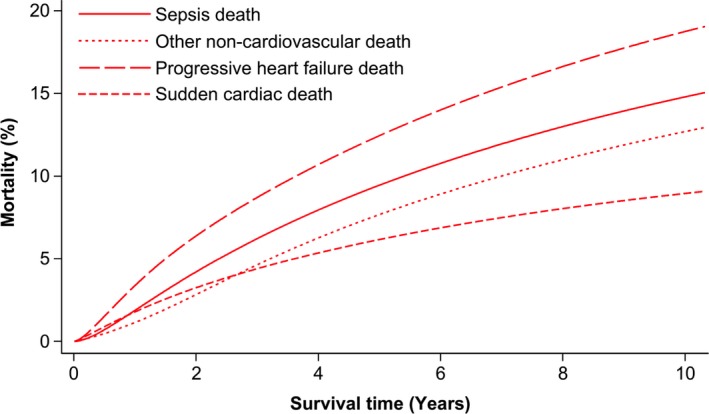
Cumulative incidence of sepsis death in relation to other common modes of death. Cumulative incidence functions illustrating sepsis, other noncardiovascular, progressive heart failure, and sudden cardiac death during follow‐up.

After a mean follow‐up period of 4 years, a total of 737 (40.9%) deaths occurred, the modes of which were progressive heart failure in 30.9%, sudden cardiac in 15.2%, other cardiovascular in 8%, noncardiovascular in 42.6%, and unclassifiable in 3.3% (Figure 1A). Within the noncardiovascular deaths, 55.1% (n=173) were due to sepsis, representing 23.5% of all deaths; the remaining noncardiovascular deaths were accounted for by cancer in 24.8% (n=78), with no other single disease process accounting for more than 5% of noncardiovascular deaths. The primary source of sepsis is illustrated in Figure 1B, with ≈70% of sepsis deaths involving the respiratory tract. The cumulative incidence of sepsis and other modes of death is illustrated in Figure 2. Notably, a sensitivity analysis restricted to people with LVEF <40% (n=1312) reached almost identical conclusions, with 22.2% of deaths being due to sepsis (Figure S1A), with ≈70% of cases involving the respiratory tract (Figure S1B), following a similar time‐course in cumulative incidence curves (Figure S2).

The recruitment characteristics of patients with sepsis‐ and non–sepsis‐related modes of death are shown in Table 1, with characteristics of survivors also presented for reference. Of patients who died, sepsis death was associated with greater age, increased platelet count, increased prevalence of COPD, and borderline lower serum vitamin D concentration (*P*=0.06). Notably, these differences were apparent even when patients with sepsis death were compared with a heterogeneous mix of nonsepsis deaths, including progressive heart failure, sudden cardiac death, and noncardiovascular deaths. We also noted that sepsis death was significantly more likely to occur in a hospital setting (86.1% [n=149]) than other deaths (49.4% [n=267]; *P*<0.001).

**Table 1:** Patient Characteristics

	Sepsis Death (n=173)	Nonsepsis Death (n=540)	All‐Cause Death (n=737)	Survivors (n=1065)
Age, y	75.2 (0.7)^a^	73 (0.4)	73.5 (0.4)^b^	67 (0.4)
Heart rate, bpm	76.8 (1.4)	74.1 (0.8)	74.8 (0.7)	75.7 (0.6)
QRS interval, ms	127 (3)	125 (1)	126 (1)^b^	121 (1)
Hemoglobin, g/dL	13 (0.1)	13 (0.1)	13 (0.1)^b^	13.8 (0.1)
WCC, ×10^9^/L	8.03 (0.19)	7.89 (0.14)	7.94 (0.11)^b^	7.64 (0.06)
Lymphocytes, ×10^9^/L	1.67 (0.09)	1.54 (0.03)	1.58 (0.03)^b^	1.82 (0.02)
Neutrophils, ×10^9^/L	5.42 (0.15)	5.37 (0.1)	5.4 (0.08)^b^	4.9 (0.05)
Platelets, ×10^9^/L	257 (6)^a^	237 (3)	243 (3)	248 (2)
Sodium, mmol/L	138.9 (0.3)	138.8 (0.2)	138.8 (0.1)^b^	139.8 (0.1)
eGFR, mL/(kg·min)	51.6 (1.4)	50.6 (0.8)	50.9 (0.7)^b^	62.5 (0.6)
Albumin, g/L	42.1 (0.2)	42 (0.2)	42 (0.1)^b^	43.6 (0.1)
Vitamin D, nmol/L	26 (16‐46)	30 (20‐47.7)	29.5 (19.8‐46.8)^b^	35 (21‐54.1)
LVEF, %	32.2 (0.7)	30.9 (0.4)	31.3 (0.4)^b^	32.5 (0.3)
Ramipril dose, mg/d	4.1 (0.3)	4.7 (0.2)	4.5 (0.1)^b^	5.2 (0.1)
Bisoprolol dose, mg/d	3 (0.2)	3.4 (0.1)	3.3 (0.1)^b^	4.3 (0.1)
Furosemide dose, mg/d	59.9 (3.4)	65.4 (2.3)	64 (1.9)^b^	42.4 (1.4)
MRA prescription, n (%)	60 (34.7)	224 (41.6)	297 (40.4)	392 (36.9)
Male sex, n (%)	133 (76.9)	424 (78.5)	577 (78.3)^b^	742 (69.7)
Diabetes mellitus, n (%)	49 (28.3)	185 (34.3)	242 (32.8)^b^	262 (24.6)
COPD, n (%)	59 (34.1)^a^	99 (18.3)	167 (22.7)^b^	117 (11)
Ischemic etiology, n (%)	111 (64.2)	373 (69.1)	503 (68.2)^b^	564 (53)
ICD (n [%])	16 (9.2)	67 (12.4)	85 (11.5)	125 (11.7)
CRT (n [%])	48 (27.7)	140 (25.9)	196 (26.6)	259 (24.3)
NYHA class				
1, n (%)	14 (8.1)	56 (10.4)	74 (10.1)^b^	259 (24.3)
2, n (%)	79 (45.7)	260 (48.3)	353 (48)	559 (52.5)
3, n (%)	77 (44.5)	207 (38.5)	290 (39.5)	244 (22.9)
4, n (%)	3 (1.7)	15 (2.8)	18 (2.4)	3 (0.3)

Data are presented as mean (standard error of the mean) or median (interquartile range) for continuous variables and as n (%) for categorical variables. COPD indicates chronic obstructive pulmonary disease; CRT, cardiac resynchronization therapy; eGFR, estimated glomerular filtration rate; ICD, implantable cardioverter defibrillator; LVEF, left ventricular ejection fraction; MRA, mineralocorticoid receptor antagonist; NYHA, New York Heart Association; WCC, white cell count.

a Sepsis vs nonsepsis death, *P*<0.05.

b All‐cause death vs survivors, *P*<0.05.

### Predictors of Sepsis Death

Unadjusted competing risk regression (sepsis death versus survival or competing causes of death) showed that advancing age, COPD, higher New York Heart Association class, lower hemoglobin, renal dysfunction, lower albumin, lower serum vitamin D, and higher diuretic requirements were each univariately associated with sepsis death (Table 2). After adjustment for all variables concurrently, advancing age (hazard ratio=1.05; 95% CI 1.03‐1.07), COPD (2.43, 1.74‐3.40), male sex (1.73; 1.16‐2.60), lower log serum vitamin D (0.68; 0.49‐0.95), and rising platelet count (1.002; 1.000‐1.005) were independently associated with increased risk of sepsis death (Table 2). Notably, a sensitivity analysis restricted to people with LVEF <40% (n=1312) reached very similar conclusions, with advancing age (hazard ratio=1.04; 95% CI 1.02‐1.07), COPD (hazard ratio=2.12; 95% CI 1.38‐3.25), and lower log serum vitamin D (hazard ratio=0.61; 95% CI 0.41‐0.92) remaining the most strongly associated variables (Table S2).

**Table 2:** Predictors of Sepsis Death

Variable	Univariate		Multivariate	
	HR (95% CI)	*P* Value	HR (95% CI)	*P* Value
Age (per y)	1.05 (1.04‐1.07)	<0.001	1.05 (1.03‐1.07)	<0.001
Male sex	1.20 (0.84‐1.72)	0.31	1.73 (1.16‐2.60)	0.008
Diabetes mellitus	1.09 (0.79‐1.52)	0.6	0.91 (0.63‐1.31)	0.611
COPD	3.07 (2.24‐4.20)	<0.001	2.43 (1.74‐3.40)	<0.001
Ischemic etiology	1.20 (0.88‐1.63)	0.258	0.78 (0.55‐1.11)	0.172
NYHA class (vs 1)		<0.001		0.079
2	2.27 (1.30‐3.98)		1.66 (0.92‐3.00)	
3	3.71 (2.12‐6.52)		2.18 (1.17‐4.07)	
4	3.32 (0.92‐12.03)		1.54 (0.39‐6.05)	
Heart rate (per bpm)	1.01 (1.00‐1.01)	0.167	1.00 (1.00‐1.01)	0.347
QRS interval (per ms)	1.00 (1.00‐1.01)	0.062	1.00 (1.00‐1.01)	0.333
Hemoglobin (per g/dL)	0.82 (0.75‐0.90)	<0.001	0.90 (0.80‐1.00)	0.052
WCC (per 10^9^/L)	1.03 (0.99‐1.06)	0.174	···	···
Lymphocytes (per 10^9^/L)	0.85 (0.60‐1.21)	0.372	1.13 (0.82‐1.58)	0.452
Neutrophils (per 10^9^/L)	1.06 (1.01‐1.12)	0.018	0.99 (0.93‐1.06)	0.784
Platelets (per 10^9^/L)	1.002 (1.00‐1.004)	0.12	1.002 (1.000‐1.005)	0.027
Sodium (per mmol/L)	0.96 (0.92‐1.00)	0.043	0.98 (0.94‐1.03)	0.383
eGFR (per mL/[kg·min])	0.98 (0.98‐0.99)	<0.001	0.99 (0.98‐1.00)	0.248
Albumin (per g/L)	0.94 (0.91‐0.97)	<0.001	0.99 (0.95‐1.03)	0.531
Vitamin D (per 2.82‐fold increase)	0.65 (0.48‐0.89)	0.008	0.68 (0.49‐0.95)	0.022
LVEF (per %)	1.000.99‐1.02)	0.691	1.00 (0.98‐1.02)	0.921
Diuretic dose (per mg/d)	1.003 (1.001‐1.005)	0.005	1.00 (1.00‐1.00)	0.886

CI indicates confidence interval; COPD, chronic obstructive pulmonary disease; eGFR, estimated glomerular filtration rate; HR, hazard ratio; LVEF, left ventricular ejection fraction; NYHA, New York Heart Association; WCC, white cell count.

**Figure 3:**
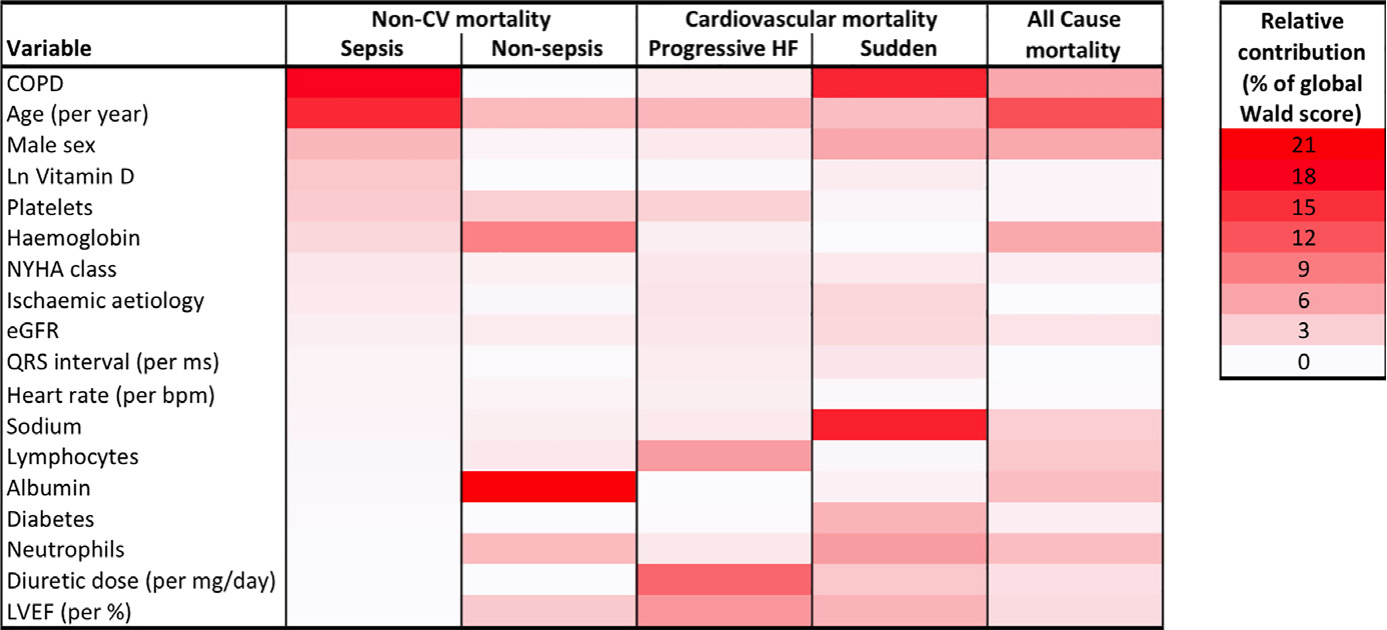
Predictors of sepsis death differ from those of alternate modes of death. Heat map of relative variable contributions to multivariate models predicting mode‐specific or all‐cause mortality, illustrating the distinct risk marker association with sepsis death. More intense red tones denote a greater percentage contribution of a variable to the global Wald score for prediction of a specific mode of death. COPD indicates chronic obstructive pulmonary disease; CV, cardiovascular; eGFR, estimated glomerular filtration rate; HF, heart failure; Ln, natural logarithm; LVEF, left ventricular ejection fraction; NYHA, New York Heart Association.

Crucially, the variables most strongly associated with sepsis death showed a distinct profile compared to other noncardiovascular death, progressive heart failure, sudden cardiac death, and all‐cause death (Figure 3 and Tables S3 through S7). Moreover, although advancing age, male sex, COPD, and higher platelet count were associated with several modes of death, low serum vitamin D showed a unique association with sepsis death; lower hemoglobin and lower albumin were uniquely associated with other noncardiovascular death; higher New York Heart Association class, lower lymphocyte count, lower LVEF, and higher diuretic dose were uniquely associated with progressive heart failure death; lower sodium and higher neutrophil counts were uniquely associated with sudden cardiac death.

## Discussion

Our analysis of a large well‐characterized cohort with CHF and reduced LVEF shows that sepsis accounts for almost a quarter of deaths, making this the second most common mode of death after progressive heart failure. We also present the first‐ever analysis of sepsis death risk markers and show that this risk marker profile is distinct from other common modes of death. Importantly, our major observations were unchanged in sensitivity analyses restricted to people with heart failure and reduced ejection fraction (HF‐REF) defined using the most contemporary definitions (ie, LVEF <40%). This may have major implications for strategies to further improve the outcomes of people with CHF and poses important questions about whether sepsis can be prevented or mitigated to improve net survival. More broadly, our analyses highlight the heterogeneous mortality of people with CHF, further emphasizing that more nuanced risk stratification and intervention may offer a more effective approach to improve overall survival.

### Sepsis and Adverse Outcomes in CHF Cohorts

Little is known about sepsis death in strictly defined populations with HF‐REF, although some data are available from mixed populations also including heart failure with preserved LVEF. Lee et al described factors underlying 1025 deaths between 1971 and 2004 in Framingham Heart Study participants with heart failure^6^; LVEF was known in 57% of cases, of which 59% had HF‐REF. Overall, 38% of deaths had a noncardiovascular mode, and the largest component of these was respiratory (infectious and noninfections) disease, although the contribution of sepsis per se was not described. Ueda et al studied 459 people admitted with acute decompensated heart failure to a single center in Japan between 2007 and 2011, 52% of whom had HF‐REF.^7^ During a mean 20‐month follow‐up period, 40% of deaths were noncardiovascular, and 19.5% (n=34) of these were due to sepsis, with this being more common in people aged >75 years. In a recent systematic review of heart failure with preserved LVEF epidemiological studies, 42% of deaths were noncardiovascular, with almost one third of these being due to sepsis.^14^

Regarding hospitalization, Logeart et al found that 27% of all acute decompensated heart failure admissions to 170 French hospitals on a single day in 2009 were precipitated by sepsis.^9^ Moreover, Alon et al found that of over 9000 people admitted to a hospital in Israel with decompensated heart failure, 38% had at least 1 decompensation precipitated by sepsis.^8^ Respiratory tract infections and bacteremia accounted for 77% of these, with risk markers for such hospitalizations including female sex, advancing age, COPD, chronic kidney disease, and anemia.

### Wider Context of Sepsis Death

Severe sepsis hospitalization rates more than doubled in the United States between 2000 and 2007,^15^ and sepsis has been estimated to contribute to between a third and a half of deaths occurring in US hospitals.^16^ CHF is an established risk marker for adverse outcomes during hospitalization with sepsis,^17^, ^18^ and so the improving survival of people with CHF is likely to increase the adverse coincidence of sepsis and CHF. Influenza and pneumococcus vaccinations are an important strategy to prevent sepsis in people with CHF,^19^, ^20^ but given the high uptake of these in UK primary care,^19^ our data may suggest that additional strategies are needed. Unfortunately, we do not have data on the provision of vaccination and the incidence of (nonfatal) sepsis in our cohort. A further window of opportunity may be during the management of early sepsis, when recognition of the adverse risk associated with CHF could prompt more intensive monitoring and treatment. However, further research is required to address these issues.

### Risk Markers of Sepsis Death

Our study is the first to define risk markers for sepsis death in unselected people with CHF. We found that a wide range of patient factors were associated with sepsis death in univariate analyses, and these broadly reflected age, heart failure severity, and comorbidity. Our multivariate analysis narrowed these to advancing age, COPD, male sex, lower vitamin D, and higher platelet count. Although advancing age is a risk marker for most adverse events, and COPD is unsurprising given that most sepsis deaths were respiratory tract in origin, the other risk markers were potentially less anticipated. Indeed, there are no data linking vitamin D status to sepsis events in people with heart failure; however, low serum vitamin D has been associated with sepsis incidence and mortality in other populations,^21^ and a recent meta‐analysis suggested that correction of vitamin D deficiency may reduce respiratory infections.^22^

### Sepsis as a Distinct Mode of Death

A key finding of our study is the distinct contribution of baseline patient characteristics to the prediction of sepsis, other noncardiovascular, progressive heart failure, and sudden cardiac death. This implies that sepsis is a distinct mode of death and not simply a misclassification of other modes of death. Perhaps more importantly, by showing unique/specific risk markers for each mode of death, our observations suggest that more nuanced risk prediction could guide targeted interventions to improve survival. However, the primary aim of these analyses was to define whether mode‐specific risk marker profiles exist, so further work will be required to understand whether this knowledge can improve care. Importantly, our data do not imply that heart failure–directed care is unimportant to improving outcomes of people most at risk of sepsis death, or vice versa; indeed, heart failure status is likely to be a critical factor in survival from severe sepsis. Instead, our observations highlight prevention and/or treatment priorities to further improve outcomes of patient groups already established on contemporary evidence‐based heart failure therapy. It is also important to clarify that our data cannot be used to infer specific subtypes of sepsis death, such as those related primarily to respiratory failure versus multiorgan failure, but it is likely that sepsis deaths are heterogeneous.

### Strengths and Limitations

Our study provides entirely novel insights regarding the prevalence, nature, and predictors of sepsis in people with CHF, with potentially important implications for how we can continue to improve survival. However, it is important to acknowledge the limitations of our study that will need to be addressed by future research. First, we cannot comment on whether increased sepsis incidence or case fatality underpins the association of our identified risk markers with sepsis death. This differentiation is important because it could inform us about the potential value of better sepsis prevention versus sepsis care. Second, the observational nature of our study prevents us from reaching causal inferences about the identified associations of risk markers. Third, it is possible that our data are not generalizable to all heart failure populations, particularly those with preserved ejection fraction, but the existing data from mixed CHF populations across the world provide some support for the high prevalence of sepsis death. Fourth, we do not have data on specific pathogens in most of our patients, reflecting the challenge in establishing this in many cases of respiratory tract infection. Finally, it remains unclear how preventing sepsis death would impact on other outcomes important to people with CHF, and valuable insights may be gained by studying changes in the quality of life of people with CHF surviving severe sepsis.

## Conclusions

Sepsis accounts for almost a quarter of deaths in people with HF‐REF receiving contemporary care, suggesting that ongoing efforts to improve survival will need to carefully consider sepsis prevention and management. Sepsis death is associated with a risk marker profile distinct from other important modes of death, suggesting that it may be amenable to prediction and prevention.

## Sources of Funding

This work was supported by the British Heart Foundation (PG/08/020/24617). Walker and Patel hold British Heart Foundation Clinical Research Training Fellowships. Paton and Gierula hold National Institute of Health Research PhD Fellowships. Witte holds a National Institute of Health Research Clinician Scientist Fellowship. Kearney is a British Heart Foundation Professor, and Cubbon is a British Heart Foundation Intermediate Clinical Fellow.

## Disclosures

Gierula has received a research grant from Medtronic. Witte has received speaker fees from Medtronic, Livanova, St. Jude Medical, Pfizer, Bayer, and BMS. Kearney has received speaker fees from Merck and NovoNordisk and unrestricted research awards from Medtronic. The remaining authors have no disclosures to report.

## Supporting information


**Data S1.** Supplemental Methods
**Table S1.** Imputation Model Specification for 20 Iterations and 50 Imputations
**Table S2.** Multivariate Predictors of Sepsis Death in People With LVEF<40%
**Table S3.** Multivariate Predictors of Sepsis Death
**Table S4.** Multivariate Predictors of Progressive Heart Failure Death
**Table S5.** Multivariate Predictors of Sudden Cardiac Death
**Table S6.** Multivariate Predictors of Other (Nonsepsis) Noncardiovascular Death
**Table S7.** Multivariate Predictors of All‐Cause Death
**Figure S1.** Sepsis death in relation to other common modes of death in people with LVEF <40%. A, Relative contribution of modes of death to overall mortality, with sepsis (represented by the hatched region) accounting for over half of noncardiovascular death. B, Relative contribution of primary sources of sepsis to overall sepsis deaths.
**Figure S2.** Cumulative incidence of sepsis death in relation to other common modes of death in people with LVEF <40%. Cumulative incidence functions illustrating sepsis, other noncardiovascular death, progressive heart failure death, and sudden cardiac death during follow‐up of people with LVEF <40%.

## References

[1] Go AS , Mozaffarian D , Roger VL , Benjamin EJ , Berry JD , Blaha MJ , Dai S , Ford ES , Fox CS , Franco S , Fullerton HJ , Gillespie C , Hailpern SM , Heit JA , Howard VJ , Huffman MD , Judd SE , Kissela BM , Kittner SJ , Lackland DT , Lichtman JH , Lisabeth LD , Mackey RH , Magid DJ , Marcus GM , Marelli A , Matchar DB , McGuire DK , Mohler ER III , Moy G , Pandey DK , Paynter NP , Reeves MJ , Sorlie PD , Stein J , Towfighi A , Turan TN , Virani SS , Wong ND , Woo D , Turner TB ; American Heart Association Statistics Committee and Stroke Statistics Subcommittee . Heart disease and stroke statistics—2014 update. Circulation. 2014;129:e28–e292. 24352519 10.1161/01.cir.0000441139.02102.80 PMC5408159

[2] Cubbon RM , Gale CP , Kearney LC , Schechter CB , Brooksby WP , Nolan J , Fox KA , Rajwani A , Baig W , Groves D , Barlow P , Fisher AC , Batin PD , Kahn MB , Zaman AG , Shah AM , Byrne JA , Lindsay SJ , Sapsford RJ , Wheatcroft SB , Witte KK , Kearney MT . Changing characteristics and mode of death associated with chronic heart failure caused by left ventricular systolic dysfunction: a study across therapeutic eras. Circ Heart Fail. 2011;4:396–403. 21562056 10.1161/CIRCHEARTFAILURE.110.959882

[3] Christiansen MN , Køber L , Weeke P , Vasan RS , Jeppesen JL , Smith JG , Gislason GH , Torp‐Pedersen C , Andersson C . Age‐specific trends in incidence, mortality, and comorbidities of heart failure in Denmark, 1995 to 2012. Circulation. 2017;135:1214–1223. 28174193 10.1161/CIRCULATIONAHA.116.025941

[4] Chen J , Normand SL , Wang Y , Krumholz HM . National and regional trends in heart failure hospitalization and mortality rates for Medicare beneficiaries, 1998–2008. JAMA. 2011;306:1669–1678. 22009099 10.1001/jama.2011.1474 PMC3688069

[5] Shen L , Jhund P , Petrie M , Claggett B , Barlera S , Cleland JGF , Dargie HJ , Granger CB , Kjekshus J , Køber L , Latini R , Maggioni AP , Packer M , Pitt B , Solomon SD , Swedberg K , Tavazzi L , Wikstrand J , Zannad F , Zile MR , McMurray JJV . Declining risk of sudden death in heart failure. N Engl J Med. 2017;377:41–51. 28679089 10.1056/NEJMoa1609758

[6] Lee DS , Gona P , Albano I , Larson MG , Benjamin EJ , Levy D , Kannel WB , Vasan RS . A systematic assessment of causes of death after heart failure onset in the community: impact of age at death, time period, and left ventricular systolic dysfunction. Circ Heart Fail. 2011;4:36–43. 21071547 10.1161/CIRCHEARTFAILURE.110.957480 PMC3243964

[7] Ueda T , Kawakami R , Horii M , Sugawara Y , Matsumoto T , Okada S , Nishida T , Soeda T , Okayama S , Somekawa S , Takeda Y , Watanabe M , Kawata H , Uemura S , Saito Y . Noncardiovascular death, especially infection, is a significant cause of death in elderly patients with acutely decompensated heart failure. J Card Fail. 2014;20:174–180. 24361802 10.1016/j.cardfail.2013.12.007

[8] Alon D , Stein GY , Korenfeld R , Fuchs S . Predictors and outcomes of infection‐related hospital admissions of heart failure patients. PLoS One. 2013;8:e72476. 24009684 10.1371/journal.pone.0072476 PMC3751916

[9] Logeart D , Isnard R , Resche‐Rigon M , Seronde MF , de Groote P , Jondeau G , Galinier M , Mulak G , Donal E , Delahaye F , Juilliere Y , Damy T , Jourdain P , Bauer F , Eicher JC , Neuder Y , Trochu JN ; Heart Failure of the French Society of Cardiology . Current aspects of the spectrum of acute heart failure syndromes in a real‐life setting: the OFICA study. Eur J Heart Fail. 2013;15:465–476. 23186936 10.1093/eurjhf/hfs189

[10] Witte KK , Patel PA , Walker AMN , Schechter CB , Drozd M , Sengupta A , Byrom R , Kearney LC , Sapsford RJ , Kearney MT , Cubbon RM . Socioeconomic deprivation and mode‐specific outcomes in patients with chronic heart failure. Heart. 2018;104:993–998. 29386325 10.1136/heartjnl-2017-312539 PMC5992368

[11] Witte KK , Drozd M , Walker AM , Patel PA , Kearney JC , Chapman S , Sapsford RJ , Gierula J , Paton MF , Lowry J , Kearney MT , Cubbon RM . Mortality reduction associated with β‐adrenoceptor inhibition in chronic heart failure is greater in patients with diabetes. Diabetes Care. 2017;41:136–142. 28982651 10.2337/dc17-1406

[12] Fine JP , Gray RJ . A proportional hazards model for the subdistribution of a competing risk. J Am Stat Assoc. 1999;94:496–509.

[13] Witte KK , Byrom R , Gierula J , Paton MF , Jamil HA , Lowry JE , Gillott RG , Barnes SA , Chumun H , Kearney LC , Greenwood JP , Plein S , Law GR , Pavitt S , Barth JH , Cubbon RM , Kearney MT . Effects of vitamin D on cardiac function in patients with chronic HF: the VINDICATE study. J Am Coll Cardiol. 2016;67:2593–2603. 27058906 10.1016/j.jacc.2016.03.508 PMC4893154

[14] Vaduganathan M , Patel RB , Michel A , Shah SJ , Senni M , Gheorghiade M , Butler J . Mode of death in heart failure with preserved ejection fraction. J Am Coll Cardiol. 2017;69:556–569. 28153111 10.1016/j.jacc.2016.10.078

[15] Kumar G , Kumar N , Taneja A , Kaleekal T , Tarima S , McGinley E , Jimenez E , Mohan A , Khan RA , Whittle J , Jacobs E , Nanchal R ; Milwaukee Initiative in Critical Care Outcomes Research (MICCOR) Group of Investigators . Nationwide trends of severe sepsis in the 21st century (2000–2007). Chest. 2011;140:1223–1231. 21852297 10.1378/chest.11-0352

[16] Liu V , Escobar G , Greene J , Soule J , Whippy A , Angus DC , Iwashyna TJ . Hospital deaths in patients with sepsis from 2 independent cohorts. JAMA. 2014;312:90–92. 24838355 10.1001/jama.2014.5804

[17] Ford DW , Goodwin AJ , Simpson AN , Johnson E , Nadig N , Simpson KN . A severe sepsis mortality prediction model and score for use with administrative data. Crit Care Med. 2016;44:319–327. 26496452 10.1097/CCM.0000000000001392 PMC4724863

[18] Aliberti S , Amir A , Peyrani P , Mirsaeidi M , Allen M , Moffett B , Myers J , Shaib F , Cirino M , Bordon J , Blasi F , Ramirez JA . Risk factors for clinical failure in hospitalized patients with community‐acquired pneumonia. Chest. 2008;134:955–962. 18583514 10.1378/chest.08-0334

[19] Vardeny O , Claggett B , Udell JA , Packer M , Zile M , Rouleau J , Swedberg K , Desai AS , Lefkowitz M , Shi V , McMurray JJV , Solomon SD ; PARADIGM‐HF Investigators . Influenza vaccination in patients with chronic heart failure: the PARADIGM‐HF trial. JACC Heart Fail. 2016;4:152–158. 26746371 10.1016/j.jchf.2015.10.012

[20] Bhatt AS , DeVore AD , Hernandez AF , Mentz RJ . Can vaccinations improve heart failure outcomes? Contemporary data and future directions. JACC Heart Fail. 2016;4:152–158. 28161238 10.1016/j.jchf.2016.12.007 PMC5336530

[21] de Haan K , Groeneveld ABJ , de Geus HRH , Egal M , Struijs A . Vitamin D deficiency as a risk factor for infection, sepsis and mortality in the critically ill: systematic review and meta‐analysis. Crit Care. 2014;18:660. 25475621 10.1186/s13054-014-0660-4 PMC4277653

[22] Martineau AR , Jolliffe DA , Hooper RL , Greenberg L , Aloia JF , Bergman P , Dubnov‐Raz G , Esposito S , Ganmaa D , Ginde AA , Goodall EC , Grant CC , Griffiths CJ , Janssens W , Laaksi I , Manaseki‐Holland S , Mauger D , Murdoch DR , Neale R , Rees JR , Simpson S Jr , Stelmach I , Kumar GT , Urashima M , Camargo CA Jr . Vitamin D supplementation to prevent acute respiratory tract infections: systematic review and meta‐analysis of individual participant data. BMJ. 2017;356:i6583. 28202713 10.1136/bmj.i6583 PMC5310969

