# Supplementary material for: Prevalence and Predictors of Sepsis Death in Patients With Chronic Heart Failure and Reduced Left Ventricular Ejection Fraction
Source: J Am Heart Assoc. 2018 Oct 4;7(20):e009684. doi: 10.1161/JAHA.118.009684 (PMC6474963; doi:10.1161/JAHA.118.009684)
Supplement: Supplementary file 1 — Data S1. Supplemental Methods Table S1. Imputation Model Specification for 20 Iterations and 50 Imputations Table S2. Multivariate Predictors of Sepsis Death in People With LVEF<40% Table S3. Multivariate Predictors of Sepsis Death Table S4. Multivariate Predictors of Progressive Heart Failure Death Table S5. Multivariate Predictors of Sudden Cardiac Death Table S6. Multivariate Predictors of Other (Nonsepsis) Noncardiovascular Death Table S7. Multivariate Predictors of All‐Cause Death Figure S1. Sepsis death in relation to other common modes of death in people with LVEF <40%. A, Relative contribution of modes of death to overall mortality, with sepsis (represented by the hatched region) accounting for over half of noncardiovascular death. B, Relative contribution of primary sources of sepsis to overall sepsis deaths. Figure S2. Cumulative incidence of sepsis death in relation to other common modes of death in people with LVEF <40%. Cumulative incidence functions illustrating sepsis, other noncardiovascular death, progressive heart failure death, and sudden cardiac death during follow‐up of people with LVEF <40%. [file JAH3-7-e009684-s001.pdf]

Supplemental Material

Data S1.

Supplemental Methods

Imputation methods

Multiple imputation by chained equations under the missing at random assumption was used to impute 50 sets of data to minimise any potential bias caused by missing data.[1-2] The imputation model specification was such that it included all variables included in the analysis model, including outcome variables (Nelson-Aalen survival estimator for each cause of death and a censoring indicator) as well as auxiliary variables as indicated in the table below. Results presented in the manuscript contain estimates averaged over 50 imputed data sets according to Rubin's rules.

Table S1. Imputation model specification for 20 iterations and 50 imputations.

Variable	Missing (%)	Type	Specification for imputation
Nelson-Aalen survival estimate (included as separate variables for each cause of death)	0	Continuous (Outcome)	Complete data - included as an auxiliary variable for survival time
Censoring indicator	0	Binary (Outcome)	Complete data - included as an auxiliary variable
Cause of death	0	Categorical	Complete data - included as an auxiliary variable
Sex	0	Binary	Complete data - included as an auxiliary variable
Diabetes	0	Binary	Complete data - included as an auxiliary variable
COPD	0	Binary	Complete data - included as an auxiliary variable
Ischaemic HF Aetiology	0	Binary	Complete data - included as an auxiliary variable
ICD	0	Binary	Complete data - included as an auxiliary variable
CRT	0	Binary	Complete data - included as an auxiliary variable
Age	0	Continuous	Linear regression
NYHA Class	0.1	Categorical	Ordinal logistic regression
Heart Rate	8.8	Continuous	Linear regression
QRS interval	8.8	Continuous	Log transformed and imputed using linear regression
Haemoglobin	0.3	Continuous	Linear regression
Sodium	0.2	Continuous	Predictive mean matching
White blood cell count	0.3	Continuous	Predictive mean matching
Lymphocytes	1.2	Continuous	Complete data - included as an auxiliary variable
Neutrophils	1.2	Continuous	Complete data - included as an auxiliary variable
Platelet count	0.3	Continuous	Complete data - included as an auxiliary variable
eGFR	0.4	Continuous	Linear regression
VitaminD	30.5	Continuous	Log transformed and imputed using linear

			regression
Albumin	3.1	Continuous	Linear regression
LV ejection fraction	2.5	Continuous	Linear regression
Ramipril Dose	0.3	Continuous	Predictive mean matching
Bisoprolol Dose	0.3	Continuous	Predictive mean matching
Furosemide Dose	0.3	Continuous	Predictive mean matching

COPD - chronic obstructive pulmonary disease; HF - heart failure; ICD - implantable cardioverter defibrillator; CRT - cardiac resynchronisation therapy; NYHA - New York heart association; eGFR - estimated glomerular filtration rate; LV - left ventricular.

Table S2. Multivariate predictors of sepsis death in people with LVEF<40%.

Variable	HR	95% CI of HR		p value	Wald	% Global Wald
		Low	High			
Age (per year)	1.04	1.02	1.07	0.000	12.66	11.29
Male sex	1.21	0.75	1.97	0.439	0.60	0.54
Diabetes	0.88	0.56	1.40	0.592	0.29	0.26
COPD	2.12	1.38	3.25	0.001	11.91	10.62
Ischaemic aetiology	0.92	0.59	1.45	0.722	0.13	0.12
NYHA class (vs 1)				0.265	1.32	1.18
2	1.90	0.81	4.47	0.140		
3	2.39	0.98	5.81	0.055		
4	1.81	0.40	8.16	0.440		
Heart rate (per bpm)	1.01	1.00	1.02	0.113	2.51	2.24
QRS interval (per ms)	1.00	1.00	1.01	0.289	1.12	1.00
Haemoglobin (per g/dl)	0.85	0.74	0.98	0.028	4.85	4.32
Lymphocytes (per 10^9/L)	1.15	0.70	1.88	0.580	0.31	0.28
Neutrophils (per 10^9/L)	0.99	0.90	1.08	0.823	0.05	0.04
Platelets (per 10^9/L)	1.00	1.00	1.00	0.231	1.43	1.28
Sodium (per mmol/L)	0.97	0.92	1.02	0.247	1.34	1.19
eGFR (per ml/Kg/min)	0.99	0.98	1.01	0.342	0.90	0.80
Albumin (per g/L)	0.97	0.92	1.02	0.269	1.22	1.09
Vitamin D (per 2.82-fold increase)	0.61	0.41	0.92	0.018	5.62	5.01
LVEF (per %)	0.99	0.97	1.02	0.640	0.22	0.20
Diuretic dose (per mg/day)	1.00	1.00	1.00	0.762	0.09	0.08

eGFR - estimated glomerular filtration rate; LVEF - left ventricular ejection fraction; COPD - chronic obstructive pulmonary disease; ICD - implantable cardioverter defibrillator; CRT - cardiac resynchronisation therapy; NYHA - New York heart association.

Table S3. Multivariate predictors of sepsis death.

Variable	HR	95% CI of HR		p value	Wald	% Global Wald
		Low	High			
Age (per year)	1.05	1.03	1.07	<0.001	23.49	15.54
Male sex	1.73	1.16	2.60	0.008	7.06	4.67
Diabetes	0.91	0.63	1.31	0.611	0.26	0.17
COPD	2.43	1.74	3.40	<0.001	26.93	17.81
Ischaemic aetiology	0.78	0.55	1.11	0.172	1.86	1.23
NYHA class (vs 1)				0.079	2.27	1.50
2	1.66	0.92	3.00	0.095		
3	2.18	1.17	4.07	0.014		
4	1.54	0.39	6.05	0.535		
Heart rate (per bpm)	1.00	1.00	1.01	0.347	0.88	0.58
QRS interval (per ms)	1.00	1.00	1.01	0.333	0.94	0.62
Haemoglobin (per g/dl)	0.90	0.80	1.00	0.052	3.78	2.50
Lymphocytes (per 10^9/L)	1.13	0.82	1.58	0.452	0.56	0.37
Neutrophils (per 10^9/L)	0.99	0.93	1.06	0.784	0.08	0.05
Platelets (per 10^9/L)	1.002	1.000	1.005	0.027	4.86	3.21
Sodium (per mmol/L)	0.98	0.94	1.03	0.383	0.76	0.50
eGFR (per ml/Kg/min)	0.99	0.98	1.00	0.248	1.33	0.88
Albumin (per g/L)	0.99	0.95	1.03	0.531	0.39	0.26
Vitamin D (per 2.82-fold increase)	0.68	0.49	0.95	0.022	5.25	3.47
LVEF (per %)	1.00	0.98	1.02	0.921	0.01	0.01
Diuretic dose (per mg/day)	1.00	1.00	1.00	0.886	0.02	0.01

eGFR - estimated glomerular filtration rate; LVEF - left ventricular ejection fraction; COPD - chronic obstructive pulmonary disease; ICD - implantable cardioverter defibrillator; CRT - cardiac resynchronisation therapy; NYHA - New York heart association.

Table S4. Multivariate predictors of progressive heart failure death.

Variable	HR	95% CI of HR		p value	Wald	% Global Wald
		Low	High			
Age (per year)	1.03	1.01	1.05	0.001	10.23	4.69
Male sex	1.32	0.95	1.85	0.101	2.68	1.23
Diabetes	1.11	0.81	1.53	0.508	0.44	0.20
COPD	0.74	0.51	1.07	0.111	2.54	1.16
Ischaemic aetiology	1.38	0.98	1.94	0.065	3.41	1.56
NYHA class (vs 1)				0.031	2.97	1.36
2	1.77	0.99	3.18	0.055		
3	2.25	1.23	4.12	0.008		
4	3.42	1.21	9.69	0.020		
Heart rate (per bpm)	0.99	0.99	1.00	0.158	1.99	0.91
QRS interval (per ms)	1.00	0.99	1.00	0.112	2.53	1.16
Haemoglobin (per g/dl)	0.93	0.85	1.03	0.176	1.83	0.84
Lymphocytes (per 10^9/L)	0.61	0.47	0.79	<0.001	14.36	6.58
Neutrophils (per 10^9/L)	1.07	0.99	1.15	0.090	2.88	1.32
Platelets (per 10^9/L)	1.00	0.99	1.00	0.013	6.17	2.83
Sodium (per mmol/L)	0.97	0.93	1.01	0.101	2.69	1.23
eGFR (per ml/Kg/min)	0.99	0.98	1.00	0.071	3.26	1.49
Albumin (per g/L)	1.00	0.96	1.04	0.878	0.02	0.01
Ln Vitamin D (per 2.82-fold increase)	0.88	0.65	1.19	0.401	0.71	0.33
LVEF (per %)	0.97	0.96	0.98	<0.001	15.29	7.01
Diuretic dose (per mg/day)	1.01	1.00	1.01	<0.001	23	10.54

eGFR - estimated glomerular filtration rate; LVEF - left ventricular ejection fraction; COPD - chronic obstructive pulmonary disease;

ICD - implantable cardioverter defibrillator; CRT - cardiac resynchronisation therapy; NYHA - New York heart association.

Table S5. Multivariate predictors of sudden cardiac death.

Variable	HR	95% CI of HR		p value	Wald	% Global Wald
		Low	High			
Age (per year)	0.98	0.97	1.00	0.085	2.96	4.13
Male sex	1.70	1.02	2.84	0.043	4.11	5.73
Diabetes	1.46	0.98	2.18	0.064	3.44	4.80
COPD	2.19	1.39	3.46	0.001	11.35	15.83
Ischaemic aetiology	1.37	0.87	2.14	0.176	1.83	2.55
NYHA class (vs 1)				0.452	0.88	1.23
2	1.51	0.83	2.77	0.181		
3	1.17	0.59	2.29	0.657		
4	1.39	0.28	6.98	0.689		
Heart rate (per bpm)	1.00	0.99	1.02	0.669	0.18	0.25
QRS interval (per ms)	1.00	1.00	1.01	0.312	1.02	1.42
Haemoglobin (per g/dl)	1.01	0.90	1.15	0.827	0.05	0.07
Lymphocytes (per 10^9/L)	0.94	0.73	1.21	0.619	0.25	0.35
Neutrophils (per 10^9/L)	1.09	1.01	1.17	0.030	4.69	6.54
Platelets (per 10^9/L)	1.00	1.00	1.00	0.561	0.34	0.47
Sodium (per mmol/L)	0.93	0.89	0.97	0.001	11.65	16.25
eGFR (per ml/Kg/min)	0.99	0.98	1.00	0.192	1.71	2.38
Albumin (per g/L)	0.98	0.93	1.03	0.441	0.59	0.82
Ln Vitamin D (per 2.82-fold increase)	0.85	0.57	1.24	0.393	0.73	1.02
LVEF (per %)	0.98	0.96	1.00	0.062	3.47	4.84
Diuretic dose (per mg/day)	1.00	0.99	1.00	0.112	2.53	3.53

eGFR - estimated glomerular filtration rate; LVEF - left ventricular ejection fraction; COPD - chronic obstructive pulmonary disease; ICD - implantable cardioverter defibrillator; CRT - cardiac resynchronisation therapy; NYHA - New York heart association.

Table S6. Multivariate predictors of other (non-sepsis) non-cardiovascular death.

Variable	HR	95% CI of HR		p value	Wald	% Global Wald
		Low	High			
Age (per year)	1.01	1.00	1.03	0.145	2.12	4.41
Male sex	1.11	0.73	1.69	0.613	0.26	0.54
Diabetes	1.02	0.70	1.48	0.914	0.01	0.02
COPD	1.01	0.64	1.58	0.978	0	0.00
Ischaemic aetiology	0.93	0.64	1.34	0.683	0.17	0.35
NYHA class (vs 1)				0.750	0.4	0.83
2	0.77	0.47	1.26	0.299		
3	0.87	0.50	1.50	0.611		
4	0.92	0.25	3.40	0.896		
Heart rate (per bpm)	1.00	0.99	1.01	0.596	0.28	0.58
QRS interval (per ms)	1.00	0.99	1.01	0.758	0.1	0.21
Haemoglobin (per g/dl)	0.89	0.80	1.00	0.042	4.13	8.58
Lymphocytes (per 10^9/L)	0.89	0.67	1.18	0.431	0.62	1.29
Neutrophils (per 10^9/L)	1.07	0.98	1.17	0.147	2.11	4.38
Platelets (per 10^9/L)	1.00	1.00	1.00	0.232	1.43	2.97
Sodium (per mmol/L)	1.02	0.97	1.06	0.505	0.44	0.91
eGFR (per ml/Kg/min)	1.00	0.99	1.01	0.468	0.53	1.10
Albumin (per g/L)	0.92	0.87	0.97	0.001	10.12	21.03
Ln Vitamin D (per 2.82-fold increase)	0.97	0.69	1.35	0.843	0.04	0.08
LVEF (per %)	1.01	0.99	1.03	0.201	1.64	3.41
Diuretic dose (per mg/day)	1.00	1.00	1.00	0.979	0	0.00

eGFR - estimated glomerular filtration rate; LVEF - left ventricular ejection fraction; COPD - chronic obstructive pulmonary disease; ICD - implantable cardioverter defibrillator; CRT - cardiac resynchronisation therapy; NYHA - New York heart association.

Table S7. Multivariate predictors of all-cause death.

Variable	HR	95% CI of HR		p value	Wald	% Global Wald
		Low	High			
Age (per year)	1.04	1.03	1.04	<0.001	62.6	12.34
Male sex	1.67	1.38	2.01	<0.001	28.6	5.64
Diabetes	1.21	1.02	1.43	0.026	4.97	0.98
COPD	1.64	1.37	1.97	<0.001	29	5.72
Ischaemic aetiology	1.01	0.85	1.20	0.896	0.02	0.00
NYHA class (vs 1)				0.002	4.86	0.96
2	1.44	1.11	1.88	0.006		
3	1.59	1.21	2.09	0.001		
4	2.36	1.37	4.04	0.002		
Heart rate (per bpm)	1.00	1.00	1.01	0.273	1.2	0.24
QRS interval (per ms)	1.00	1.00	1.00	0.988	0	0.00
Haemoglobin (per g/dl)	0.87	0.83	0.92	<0.001	28.56	5.63
Lymphocytes (per 10^9/L)	0.77	0.68	0.87	<0.001	17.73	3.50
Neutrophils (per 10^9/L)	1.09	1.05	1.13	<0.001	21.51	4.24
Platelets (per 10^9/L)	1.00	1.00	1.00	0.093	2.82	0.56
Sodium (per mmol/L)	0.96	0.94	0.98	<0.001	15.29	3.01
eGFR (per ml/Kg/min)	0.99	0.99	1.00	0.004	8.26	1.63
Albumin (per g/L)	0.95	0.93	0.97	<0.001	21.24	4.19
Ln Vitamin D (per 2.82-fold increase)	0.88	0.76	1.02	0.100	2.72	0.54
LVEF (per %)	0.99	0.98	0.99	0.001	10.96	2.16
Diuretic dose (per mg/day)	1.00	1.00	1.00	0.002	9.48	1.87

eGFR - estimated glomerular filtration rate; LVEF - left ventricular ejection fraction; COPD - chronic obstructive pulmonary disease; ICD - implantable cardioverter defibrillator; CRT - cardiac resynchronisation therapy; NYHA - New York heart association.

Figure S1. Sepsis death in relation to other common modes of death in people with LVEF<40%.

A) Relative contribution of modes of death to overall mortality, with sepsis (represented by the hatched region) accounting for over half of non-cardiovascular death. B) Relative contribution of primary sources of sepsis to overall sepsis deaths.

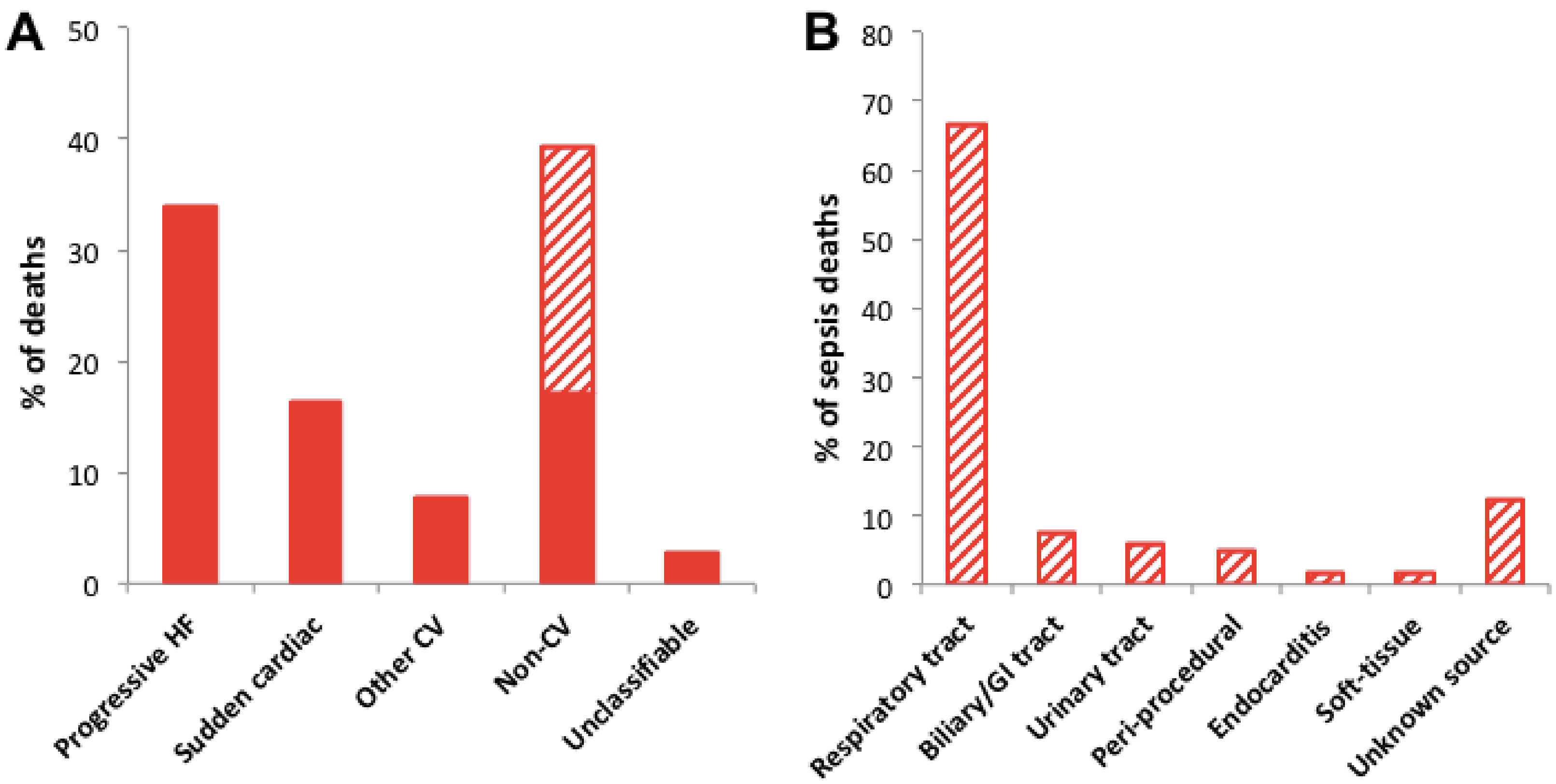

Figure S2. Cumulative incidence of sepsis death in relation to other common modes of death in people with LVEF<40%.

Cumulative incidence functions illustrating sepsis, other non-cardiovascular, progressive heart failure and sudden cardiac death during follow-up of people with LVEF<40%.

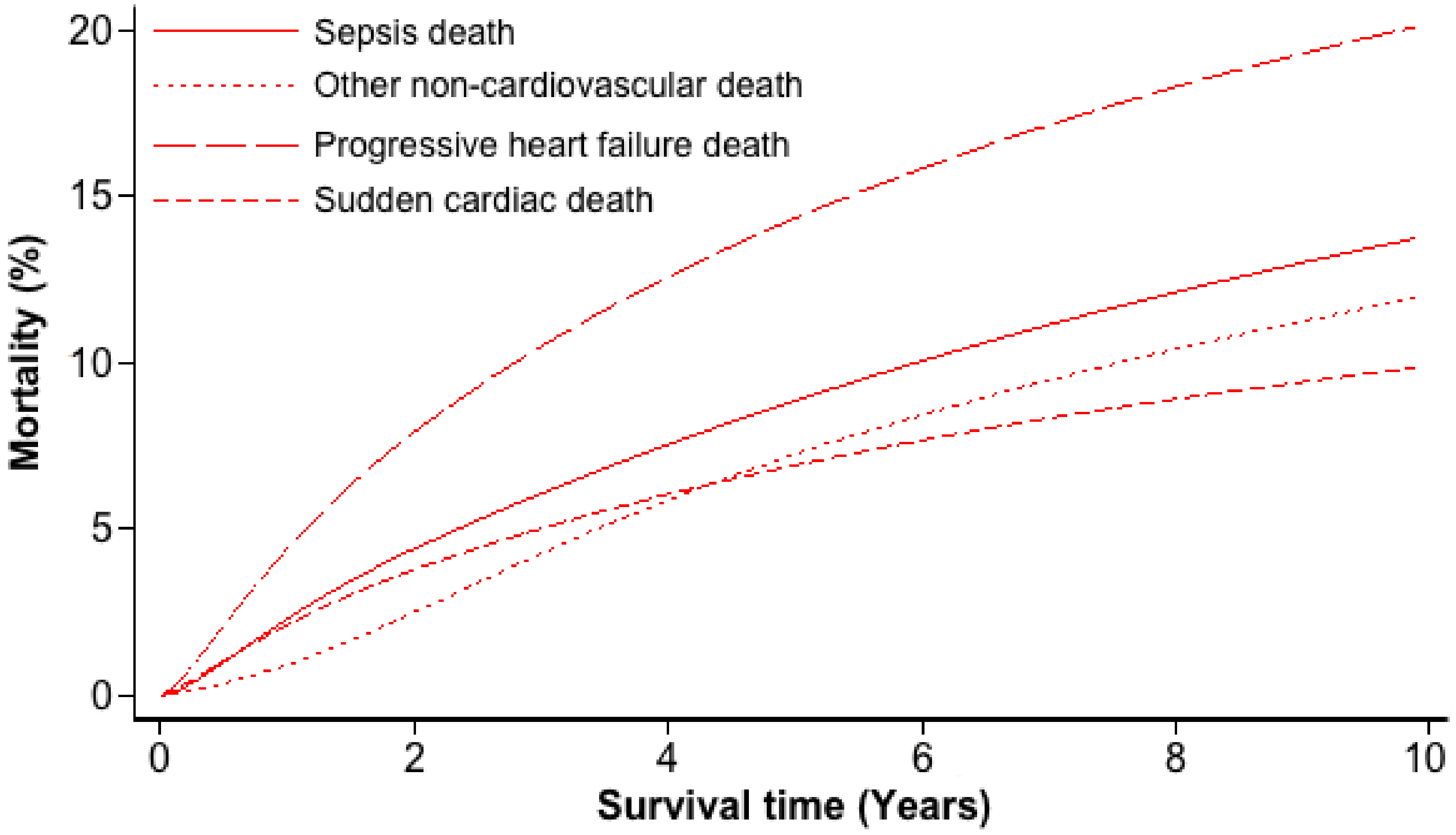

Supplemental References:

1. White IR, Royston P, Wood AM. Multiple imputation using chained equations: issues and guidance for practice. *Stat Med.* 2011;30:377-99.

2. Sterne JA, White IR, Carlin JB, Spratt M, Royston P, Kenward MG, Wood AM, Carpenter JR. Multiple imputation for missing data in epidemiological and clinical research: potential and pitfalls. *BMJ*. 2009;338:b2393.
